# Revisiting neuropsychiatry as a psychiatric discipline

**DOI:** 10.1192/bjb.2018.47

**Published:** 2018-08

**Authors:** Jack C. Lennon

**Affiliations:** Department of Clinical Neuropsychology, Illinois School of Professional Psychology, USA; email: jlennon11@live.com

There has been a long-standing debate around the merging of disciplines, owing not to a lack of necessity in multidisciplinary approaches, but the increase in specialisation and fear of each field losing its perceived uniqueness. Fitzgerald^1^ made this clear in his remarks regarding how neuropsychiatry can possess that which is both neurology and psychiatry. In a strong refutation, Datta^2^ stated with vigour that while psychiatry could be improved with greater knowledge of behavioural neurology and neurosciences, it is unique in its approach. This rebuttal seems to have touched on a view of the psyche that may be one specific to psychiatry and psychology, but it cannot escape the organ from which these cognitions spontaneously arise.

It is clear that behavioural neuroscience is the foundation of psychiatry, an unassailable statement with which Datta^2^ agreed. Fitzgerald's editorial,^1^ however, does not necessarily minimise or underappreciate the importance of psychiatry in managing presentations such as suicidality from psychosocial perspectives, which a neurologist, for example, would not have the training to assess and treat. Instead, this editorial seems to serve an explicit purpose – to identify how specialisation in these specific circumstances may actually harm the field of psychiatry. It can be seen in the present day that psychiatry is stigmatised as much as the disorders it treats – it has been viewed as an intentional deviation from neurology, which is well known to treat organic disorders of the brain. A lack of merger, then, serves to ostracise psychiatry insofar that it becomes less about the brain. To state that discussions about psychotherapy and other psychological interventions are not rooted in neuroscience would be demonstrably fallacious. Even cognitive–behavioural therapy, one of the most studied psychotherapeutic interventions, was developed by a neurologist and recognises that the brain undergoes significant changes due to cognitions, ultimately leading to maladaptive behaviours beyond conscious awareness or personal agency.^3^ One cannot speak of psychiatric disorders without recognising and appreciating the organ responsible for behaviour and cognition.

The formulation of diagnoses and treatments for psychiatric conditions certainly differs from that of ‘neurological’ conditions such as mild traumatic brain injury, stroke and epilepsy. However, significant evidence suggests that psychiatric research is on the brink of discovering quantitative measures of its disorders, including but not limited to neuroimaging techniques,^4^^,^^5^ neuropsychological evaluations,^6^^–^^8^ and psychological tests such as those related to implicit associations.^9^^,^^10^ If one is to believe that psychiatry will remain entirely distinct from neurology and that the field need not merge with the professionals who also treat the central nervous system manifestations of brain dysfunction, regardless of aetiology, then psychiatry is destined to lose its footing when technology catches up to the incessantly changing brain that falls prey to inter- and intrapersonal events.

## References

[1] Fitzgerald M. Do psychiatry and neurology need a close partnership or merger? BJPsych Bull 2015; 39(3): 105–7. 26191446 10.1192/pb.bp.113.046227 PMC4478930

[2] Datta V. Psychiatry is more than neuropsychiatry. BJPsych Bull 2015; 39(5): 263. doi:10/1192/pb.39.5.263 10.1192/pb.39.5.263 PMC4706188 26755979

[3] Beck JS, Beck AT. Cognitive Behavior Therapy: Basics and Beyond (2nd edn). Guilford Press, 2011.

[4] Zhang H, Chen Z, Jia Z, Gong Q. Dysfunction of neural circuitry in depressive patients with suicidal behaviors: a review of structural and functional neuroimaging. Prog Neuropsychopharmacol Biol Psychiatry 2014; 53: 61–6. 24632395 10.1016/j.pnpbp.2014.03.002

[5] Sublette ME, Milak MS, Galfalvy HC, Oquendo MA, Malone KM, Mann JJ. Regional brain glucose uptake distinguishes suicide attempters from non-attempters in major depression. Arch Suicide Res 2013; 17(4): 434–47. 24224676 10.1080/13811118.2013.801813 PMC3831169

[6] Gujral S, Dombrovski AY, Butters M, Clark L, Reynolds III CF, Szanto K. Impaired executive function in contemplated and attempted suicide in late life. Am J Geriatr Psychiatry 2014; 22(8): 811–9. 23567385 10.1016/j.jagp.2013.01.025 PMC3516623

[7] Keilp JG, Gorlyn M, Russell M, Oquendo MA, Burke AK, Harkavy-Friedman J, Neuropsychological function and suicidal behavior: attention control, memory and executive dysfunction in suicide attempt. Psychol Med 2013; 43(3): 539–51. 22781400 10.1017/S0033291712001419 PMC3767483

[8] Richard-Devantoy S, Berlim MT, Jollant F. A meta-analysis of neuropsychological markers of vulnerability to suicidal behavior in mood disorders. Psychol Med 2014; 44(8): 1663–73. 24016405 10.1017/S0033291713002304

[9] Nock MK, Park JM, Finn CT, Deliberto TL, Dour HJ, Banaji MR. Measuring the suicidal mind: implicit cognitions predict suicidal behavior. Psychol Sci 2010; 21(4): 511–7. 20424092 10.1177/0956797610364762 PMC5258199

[10] Glenn JJ, Werntz AJ, Slama SJK, Steinman SA, Teachman BA, Nock MK. Suicide and self-injury-related implicit cognition: a large-scale examination and replication. J Abnorm Psychol 2017; 126(2): 199–211. 27991808 10.1037/abn0000230 PMC5305619

